# Supplementary material for: Association between gastroesophageal reflux disease and dental caries among adults in the Azar cohort population: A cross-sectional study
Source: PLoS One. 2023 Aug 10;18(8):e0289802. doi: 10.1371/journal.pone.0289802 (PMC10414603; doi:10.1371/journal.pone.0289802)
Supplement: Attachment — Submitted filename: Response to Reviewers letter.doc [file pone.0289802.s002.doc]

**Response to Reviewers letter**

**Comment: We recognize that you obtained ethical approval from your local ethics committee after you began the study: your ethics approval document is dated 20, April 2022, while participants were recruited beginning in January 2022. For human subjects research, PLOS ONE requires that authors obtain ethics committee approval before beginning the study, and we do not accept retrospective ethical approval.**

Thank you for your accurate review. Unfortunately, an inadvertent error from the correspondence author led to the rejection of the manuscript. The problem mentioned in the previous decision letter has been corrected in the new version of the manuscript. We hope the manuscript will be reviewed again for publication in the journal.

The present study is a secondary analysis of the data from the baseline phase of the original study of the Azar cohort. To be more precise, our study was a study on the data extracted from the Azar cohort study. We first requested the Azar Cohort Center whether it is possible to access data and conduct this study. The reply date of the Azar Cohort Center was written as the date of recruiting subjects. It would have been more accurate if the date of access to data was inserted as the date of participant recruitment. The Azar Cohort Center provided access to the data for us, after obtaining the ethics approval.

We apologize for the inadvertent mistake, and on behalf of all the authors, I assure you that nothing unethical was done in the process of conducting the study. We respected the confidentiality of information in all stages.
