# Supplementary material for: Association between gastroesophageal reflux disease and dental caries among adults in the Azar cohort population: A cross-sectional study
Source: PLoS One. 2023 Aug 10;18(8):e0289802. doi: 10.1371/journal.pone.0289802 (PMC10414603; doi:10.1371/journal.pone.0289802)
Supplement: Attachment — Submitted filename: review.docx [file pone.0289802.s003.docx]

**Title:** It is limited, as there are many other factors that have been checked other than GERD. So you need to modify your title accordingly.

**Abstract:**

- Objectives: There are many other objectives that were not mentioned.

- Materials and methods: very confusing, as I don’t understand if all your data obtained from dataset

previously recorded, Or the clinical examination and the interviews were conducted as part of this

study. This same comment is applied to methodology section that needs to be re-written.

**Introduction:**

Line 53: move word ‘still’ after ‘are’.

Line 60: consistency in writing word ‘socioeconomic’.

Next to first paragraph, additional paragraph should be added to briefly preset the socioeconomic and environmental factors that is related or may affect dental caries.

Line 78: Add reference.

Add null hypothesis to the last paragraph

Methods:

-Very confusing as mentioned previously, which data that have been obtained from previous records and which data were obtained by clinical examination and questionnaires in this study??

-And if questionnaires were used, you have to specify is it online , face to face interview, paper based?

-What was the response rate in each questionnaire, number of items in each questionnaire, how these items were validated, any piloting for the questionnaire was done?

-Why 905 were selected from 2010 patients with GERD, and how these patients were selected.

-GERD patients only were diagnosed based on the two questions that have been mentioned.

- What do you mean by oral examiner, this is not acceptable description.

- Variables and data measurement section is not clear and more details should be added.

- Line 134-136: What are the categories?

Tables:

-Wealth index: what are the ranges of poor, moderate ….by numbers?

- Footnotes should be added to your tables

-

Discussion:

Line 202: Add few studies as you have mentioned in the text.

Line 208, 209: rephrase the sentence using DMFT term.

Line 223: Add reference

Line 231: Reference 33, 34 are irrelevant

Line 242: Discuss further regarding developing country and provide reference.

Line 253: Few studies… (however one reference was mentioned)

Line 258: Add reference

Line 258-260: The discussion is weak regarding the tobacco consumption

Discussion section needs improvement and more details and justifications for your findings should be presentd.

General comments:

English language needs to be checked.

It is nice to see at least one figure.

References need to be checked as many sentences need referencing, and some references are not really relevant to the text.
