# Supplementary material for: Association between gastroesophageal reflux disease and dental caries among adults in the Azar cohort population: A cross-sectional study
Source: PLoS One. 2023 Aug 10;18(8):e0289802. doi: 10.1371/journal.pone.0289802 (PMC10414603; doi:10.1371/journal.pone.0289802)
Supplement: Attachment — Submitted filename: Response to Reviewers-Mahboobi.doc [file pone.0289802.s004.doc]

**Response to Reviewers:**

**Reviewer 1:**

**Introduction**

- A major drawback of this manuscript is that the authors failed to review literature on GERD and dental caries. In addition, they didn’t identify any gap/s in literature that their study would want to address.

**Response:** The text was edited as suggested by the reviewer.

Page 3, Lines 64­­­­–70; Page 5, Lines 92­­­­–100.

- Generally, the paragraphs don’t flow into each other – the previous paragraph doesn’t flow to the next.

**Response:** The text editing has been performed as suggested by the reviewer.

**Methods**

- In addition to other confounding variables, the authors should have considered the oral hygiene status of the participants.
- Similarly, there is no information on the fluoride exposure of the participants in the study.

**Response:** The present study is a subsample of the data of an epidemiological study with a sample size of 15,000 people (Azar Cohort Study). Due to the large sample size, some variables, such as oral hygiene status and fluoride intake, were not investigated.

Also, due to the inclusion criteria of the Azar Cohort Study— permanent residence in the Shabestar district—it can be concluded that they were exposed to fluoride in the same conditions.

- Although information on free sugar consumption was obtained, the frequency of free sugar consumption between meals will be more appropriate in relation to caries formation.

**Response:** Based on studies by Sheiham (2001) and Moynihan (2014), both the frequency of consumption and the total amount of free sugars are important in the etiology of caries. Considering the common risk factor approach, in the present study, the amount of free sugars intake was used instead of the frequency of consumption.

*A. Sheiham. Dietary effects on dental diseases. Public Health Nutrition. 2001.4(2B),569-591.*

*P.J. Moynihan and S.A.M. Kelly, Effect on Caries of Restricting Sugars Intake: Systematic Review to Inform WHO Guidelines. Journal of Dental Research. 2014. 93(1):8-18.*

- Classification of GERD frequency of regurgitation should be referenced.

**Response:** It was added on page 8, line 164.

According to the classification of Wenzl 2020 (Never, Less than weekly, Weekly, Daily) and considering the data distribution in the sample, it was classified into three groups.

*E.M. Wenzl, R. Ried, A. Borenich, W. Petritsch, H.H. Wenzl. Low prevalence of gastroesophageal reflux symptoms in vegetarians. Indian Journal of Gastroenterology.*

GERD comprises a broad spectrum of disorders. The typical symptoms of GERD in adult patients are retrosternal or sub-sternal burning, regurgitation, epigastric pain, and dysphagia. Symptoms related to the reflux of gastric contents into the esophagus – principally heartburn and regurgitation – are widespread in the general population. Regurgitation, the perception of gastric contents moving from the stomach into the esophagus, experienced by 40–50% of patients with reflux disease, is also a typical symptom.

*R. JONES & J. P. GALMICHE. Review: What do we mean by GERD? Definition and diagnosis. Alimentary pharmacology & therapeutics. 2005; 22 (Suppl. 1): 2–10.*

**Results**

- It would have been better to compare the mean DMFT scores of both groups based on the different variables (socio-demographic, oral health practices and BMI) before the regression analysis.

**Response:** Table 2 was added according to the reviewer's suggestion.

Page 12, Line 235.

**Discussion**

- This is inadequate- the authors didn’t discuss the reasons behind their findings.

**Response:** The discussion section was expanded, and more details and justifications were discussed.

Page 15, Lines 267–272; Page 15, Lines 281–283; Pages 15 & 16, Lines 291–294; Page 16, Lines 311–313.

**Reviewer 2:**

**Title**

- It is limited, as there are many other factors that have been checked other than GERD. So you need to modify your title accordingly.

**Response:** According to the primary objective of this study, the main independent variable that might affect dental health was GERD. The variables potentially affecting this relationship, considering confounding effects, include demographic factors (age and gender), socioeconomic status (wealth score index and educational level), behavioral habits (tobacco use, alcohol consumption, frequency of toothbrushing, and consumption of sweet foods and beverages containing free sugars), and BMI.

**Abstract**

- Objectives: There are many other objectives that were not mentioned.

**Response:** According to the primary objective of this study, the main independent variable that might affect dental health was GERD. The other variables potentially affecting this relationship, considering confounding effects, include: demographic factors (age and gender), socioeconomic status (wealth score index and educational level), behavioral habits (tobacco use, alcohol consumption, frequency of toothbrushing, and consumption of sweet foods and beverages containing free sugars), and BMI.

- Materials and methods: very confusing, as I don’t understand if all your data obtained from dataset previously recorded, Or the clinical examination and the interviews were conducted as part of this study. This same comment is applied to methodology section that needs to be re-written.

**Response:** The “Methods” section was rewritten for more clarity. All data used in the present study was obtained from the dataset of ACS and had been recorded previously.

**Introduction**

- Line 53: move word ‘still’ after ‘are’.

**Response:** The text was edited as suggested by the reviewer.

Page 3, Line 55.

- Line 60: consistency in writing word ‘socioeconomic’.

**Response:** The text was edited as suggested by the reviewer.

Page 3, Line 62.

- Next to first paragraph, additional paragraph should be added to briefly preset the socioeconomic and environmental factors that is related or may affect dental caries.

**Response:** The text was edited as suggested by the reviewer.

Page 3 & 4, Lines 62–70.

- Line 78: Add reference.

**Response:** It was added.

Page 4, Line 88.

- Add null hypothesis to the last paragraph.

**Response:** It was added.

Page 5, lines 103–104.

**Methods**

- Very confusing as mentioned previously, which data that have been obtained from previous records and which data were obtained by clinical examination and questionnaires in this study? (This same comment is applied to methodology section that needs to be re-written).

**Response:** The “Methods” section was rewritten for more clarity. All the data used in this analytical cross-sectional study were obtained from the enrollment phase of the ACS and had been recorded previously.

- And if questionnaires were used, you have to specify is it online, face to face interview, paper based?

**Response:** It was addressed on page 6, Line 115.

Four questionnaires (one general, two medical, and one nutrition) consisting of 482 items were used to collect information in the Azar Cohort Study; and face-to-face interviews and clinical examinations were used to complete these questionnaires. Data are recorded online and stored in a centralized database and in 3 other locations, being backed up every 30 minutes.

- What was the response rate in each questionnaire, number of items in each questionnaire, how these items were validated, any piloting for the questionnaire was done?

**Response:** Since face-to-face interviews and clinical examinations were used to complete these questionnaires and the information was registered online in the cohort study databank, the response rate was 100%. All questionnaires are checked for completeness by field supervisors. It was addressed on page 6, Lines 115–118.

The Azar Cohort Study was set up in three phases: (i) pilot study; (ii) enrolment of participants; and (iii) regular follow-up of subjects for 15 years (Farhang 2019). The aim of the pilot phase was to appraise the feasibility of the study and reveal any unmet needs for undertaking the full-scale AZAR cohort study. Specifically, the pilot phase aimed to implement valid and reproducible methods and test the structured questionnaires. Also, the items in the questionnaires were categorized and presented in their own subgroups and were not numbered.

*Poustchi H, Eghtesad S, Kamangar F, Etemadi A, Keshtkar AA, Hekmatdoost A, et al. Prospective Epidemiological Research Studies in Iran (the PERSIAN Cohort Study): Rationale, Objectives, and Design. Am J Epidemiol. 2018;187(4):647-655.*

*Farhang S, Faramarzi E, Amini Sani N, Poustchi H, Ostadrahimi A, Alizadeh BZ, Somi MH. Cohort Profile: The AZAR cohort, a health-oriented research model in areas of major environmental change in Central Asia. Int J Epidemiol. 2019; 48(2):382-382h.*

- Why 905 were selected from 2010 patients with GERD, and how these patients were selected.

**Response:** According to the exclusion criteria, subjects with full dentures were excluded from the present study.

It was addressed on page 6, Line 133.

A total of 2010 patients with GERD were identified in the Azar cohort population, and 905 subjects without full dentures qualified for the case group. Fig 1 presents the selection process of the study subjects.

- GERD patients only were diagnosed based on the two questions that have been mentioned.

**Response:** The present study is a subsample of the data of a large epidemiological study with a sample size of 15,000 people (Azar Cohort Study). Due to the large sample size, data were collected by questionnaires.

GERD comprises a broad spectrum of disorders. The typical symptoms of GERD in adult patients are retrosternal or sub-sternal burning, regurgitation, epigastric pain, and dysphagia. Regurgitation, the perception of gastric contents moving from the stomach into the esophagus, experienced by 40–50% of patients with reflux disease, is also a typical symptom (Jones 2005).

*R. JONES & J. P. GALMICHE. Review: What do we mean by GERD? Definition and diagnosis. Alimentary pharmacology & therapeutics. 2005; 22 (Suppl. 1): 2–10.*

- What do you mean by oral examiner? This is not acceptable description.

**Response:** The text was edited as suggested by the reviewer.

Page 7, Line 152.

- Variables and data measurement section is not clear and more details should be added.

**Response:** The “Variables and data measurement” section was rewritten, and some details were added for more clarity.

Page 7 & 8, Lines 149–177.

- Line 134-136: What are the categories?

**Response:** It was addressed in the text.

Page 8, Lines 174–175.

**Tables**

- Wealth index: what are the ranges of poor, moderate ….by numbers?

**Response:** It has been addressed in the text.

Page 8, Lines 169–171.

- Footnotes should be added to your tables

**Response:** The tables were revised as suggested by the reviewer.

Pages 10, 12 & 13.

**Discussion**

- Line 202: Add few studies as you have mentioned in the text.

**Response:** It was added.

Page 14, Line 245.

- Line 208, 209: rephrase the sentence using DMFT term.

**Response:** The text was edited as suggested by the reviewer.

Page 14, Line 252.

- Line 223: Add reference

**Response:** It was added.

Page 15, Line 267.

- Line 231: Reference 33, 34 are irrelevant.

**Response:** The references were revised as suggested by the reviewer.

Page 15, Line 280.

- Line 242: Discuss further regarding developing country and provide reference.

**Response:** It was addressed on page 15 & 16, Lines 291–294.

- Line 253: Few studies… (However one reference was mentioned)

**Response:** The reference provided for this section is a systematic review study to Inform WHO Guidelines and was published in 2014. It is mentioned in this article that “The majority of studies identified were conducted on children, while only four studies were on adults".

*Moynihan PJ, Kelly SA. Effect on caries of restricting sugars intake: systematic review to inform WHO guidelines. J Dent Res. 2014;93(1):8-18.*

- Line 258: Add reference

**Response:** It was added on page 16, line 311.

According to the Fejerskov and Manji model for caries causation, at the individual level and in the presence of adequate saliva, optimum fluoride exposure and nutritional pattern are important determining factors for the development of caries.

- Line 258-260: The discussion is weak regarding the tobacco consumption.

**Response:** The text was revised as suggested by the reviewer.

Page 16, Lines 311–312

- Discussion section needs improvement and more details and justifications for your findings should be presented.

**Response:** The discussion section was expanded, and the reasons behind the findings were discussed.

**General comments**

- English language needs to be checked.

**Response:** The English text was edited professionally.

- It is nice to see at least one figure.

**Response:**It was added on page 7, Line 140.

- References need to be checked as many sentences need referencing, and some references are not really relevant to the text.

**Response:**References were revised and irrelevant references were deleted.
