# Supplementary material for: Association between gastroesophageal reflux disease and dental caries among adults in the Azar cohort population: A cross-sectional study
Source: PLoS One. 2023 Aug 10;18(8):e0289802. doi: 10.1371/journal.pone.0289802 (PMC10414603; doi:10.1371/journal.pone.0289802)
Supplement: Attachment — Submitted filename: Response to Reviewers-Mahboobi.doc [file pone.0289802.s006.doc]

**Response to Reviewers:**

**Reviewer 2:**

1. *What do you mean by proximal and distal determinants?*

**Response:** “The chain of events leading to an adverse health outcome can be both proximal and distal; proximal factors act directly or almost directly to cause diseases, while distal factors are further back in the causal chain and act via a number of intermediary causes.”

*Petersen PE. Sociobehavioural risk factors in dental caries – international perspectives. Community Dent Oral Epidemiol 2005; 33: 274–9.*

2. *Add reference (page 4, line 65).*

**Response:** The content of this paragraph is from chapter 4 of the book "*Dental Caries: the disease and its clinical management*" which is about the epidemiology of dental caries. The book has been referenced at the end of the paragraph.

3. *Re-phrase, it is not clear.*

**Response:** It was re-phrased (page 4, line 67)

These conditions provide the opportunities for and limit individual behaviors**.**

4. *What are these numbers, which currency? (Line 169)*

**Response:** It’s not a currency. The Wealth Score Index (WSI) in the Azar Cohort Study was based on assets such as dishwashers, vehicles, and flat-screen TVs as well as house conditions (e.g. the number of rooms, and type of ownership). This index was determined via Multiple Correspondence Analysis (MCA) and grouped into five quintiles, the first being the lowest and the fifth being the highest score.

**Reviewer 4:**

1. ***Introduction:***

*The introduction gives a clear context and rationale for the research. Given that tooth erosion is a prevalent issue in GERD patients, it effectively emphasizes the necessity of knowing the relationship between GERD and dental caries. The introduction also discusses the existing literature gap about the relationship between GERD and dental caries.* *However, at the end of the introductory section, a more clear and focused statement of the study's aims would be beneficial with clear hypothesis*.

**Response:** It was addressed on page 5, line 101:

This study hypothesizes that there is no association between having GERD and dental caries development considering potential confounders.

2. ***Methods:***

*The methods section discusses the study's design, participant selection, and data collection procedures in detail. Given its widespread usage in dentistry research, the use of the DMFT index as a measure of dental caries is justified. However, some small clarifications are required. For example, more information on how GERD was assessed, such as the questions or criteria utilized in the questionnaire, would be beneficial. The limitations of self-reported data, as well as their potential impact on the study's conclusions, should also be addressed.*

**Response:** It was done; page 7, lines 148-150.

- Among the variables of the Azar cohort study used in the present study, dental caries and body mass index (BMI) were measured based on clinical examinations by trained examiners. Dental caries was recorded as the number of decayed, missing, and filled teeth (DMFT index) according to WHO criteria [27], and the examiner was uninformed of the systemic conditions of the participants.

Other variables of the present study, including having GERD, were determined using a questionnaire and based on the participants' self-report (It has been addressed on pages 7 & 8; lines 152-161). The criterion for having GERD was the answer to these two questions: “Have you ever been diagnosed with gastroesophageal reflux disease?” and “Have you had reflux of food from the stomach to the esophagus in the past year?” (It has been addressed on page 6; lines 126-129).

The limitations of the study design and self-reported data have been considered in *Discussion* section (page 17, lines 324-329).

3. ***Results:***

*The results section is well-organized and presents the study's findings clearly. The statistical analysis, including regression analysis, strengthens the study. However, for significant findings, it would be beneficial to provide the effect sizes to provide a better understanding of the magnitude of the associations observed.*

**Response:** It was addressed on page 11; line 223.

4. ***Discussion:***

*The discussion section explores the study's findings in depth in relation to previous studies, noting both consistent and contradictory findings. The discussion is enriched by the inclusion of numerous aspects that may contribute to the association between GERD and dental caries, such as salivary flow, swallowing difficulty, and oral hygiene behaviors. However, a more extensive examination of the potential processes behind the observed relationships, particularly those related to the decline in cariogenic bacteria despite lower saliva quality and quantity, would be beneficial. Furthermore, the study's limitations, such as its cross-sectional design and dependence on self-reporting, are noted correctly. The conclusion emphasizes the need for additional research, which is appropriate.*

**Response:** It was done; page 15, lines 265-267.

Filipi explained that although *S. mutans* can survive in pH values <4.2, the pH decline in the oral cavity of GERD might be so remarkable that it can stop the metabolic activity of *S. mutans*.

**With best regards and thanks for the careful review and valuable comments of the reviewers.**
